# A Rare Case of Immune-Mediated Primary Adrenal Insufficiency With Cytotoxic T-Lymphocyte Antigen-4 Inhibitor Ipilimumab in Metastatic Melanoma of Lung and Neck of Unknown Primary

**DOI:** 10.7759/cureus.8602

**Published:** 2020-06-13

**Authors:** Salem Gaballa, Kyaw M Hlaing, Nathan Mahler, Safa Moursy, Ameenjamal Ahmed

**Affiliations:** 1 Internal Medicine, LewisGale Medical Center, Salem, USA

**Keywords:** immune-related adverse events, immune-checkpoint inhibitors, ipilimumab, nivolumab, primary adrenal insufficiency, metastatic melanoma

## Abstract

Immunotherapy with checkpoint inhibitors such as ipilimumab, a cytotoxic T-lymphocyte antigen-4 (CTLA-4) inhibitor, and nivolumab, a programmed death-1 (PD-1) inhibitor, has significantly improved the survival of patients with metastatic melanoma. The immune-related endocrinopathies of these treatments have been well documented, such as hypothyroidism, hyperthyroidism, primary adrenal insufficiency (PAI), insulin-dependent diabetes, and hypophysitis. We report the onset of PAI in a patient with metastatic melanoma to the lung and neck of unknown primary origin who was treated with ipilimumab. The patient's symptoms resolved with steroid replacement. After the completion of 16 cycles of another checkpoint inhibitor, nivolumab, full remission was achieved.

## Introduction

The evolution of immunotherapy in the treatment of cancer has created a paradigm shift within oncology, which is characterized by therapeutic agents being used to target immune cells rather than cancer cells. The use of checkpoint inhibitors such as ipilimumab, a cytotoxic T-lymphocyte antigen-4 (CTLA-4) inhibitor, and nivolumab, a programmed death-1 (PD-1) inhibitor, has led to long-lasting tumor responses. However, by unbalancing the immune system, these new immunotherapies also generate dysimmune toxicities, called immune-related adverse events (irAEs) that can potentially affect any tissue, including thyroid, adrenal, and pituitary gland [1]. Given their undisputed clinical efficacy, anti-CTLA-4 and anti-PD-1 antibodies are entering the routine oncological practice, and the number of patients exposed to these drugs will soon increase dramatically. Ipilimumab-associated primary adrenal insufficiency (PAI) is rare, as there have been only a few case reports about it. In this article, we discuss the presentation and management of this rare entity. Steroids can be used to treat ipilimumab-associated PAI, but the associated immunosuppression may compromise the antitumor response [2].

## Case presentation

**Figure 1:**
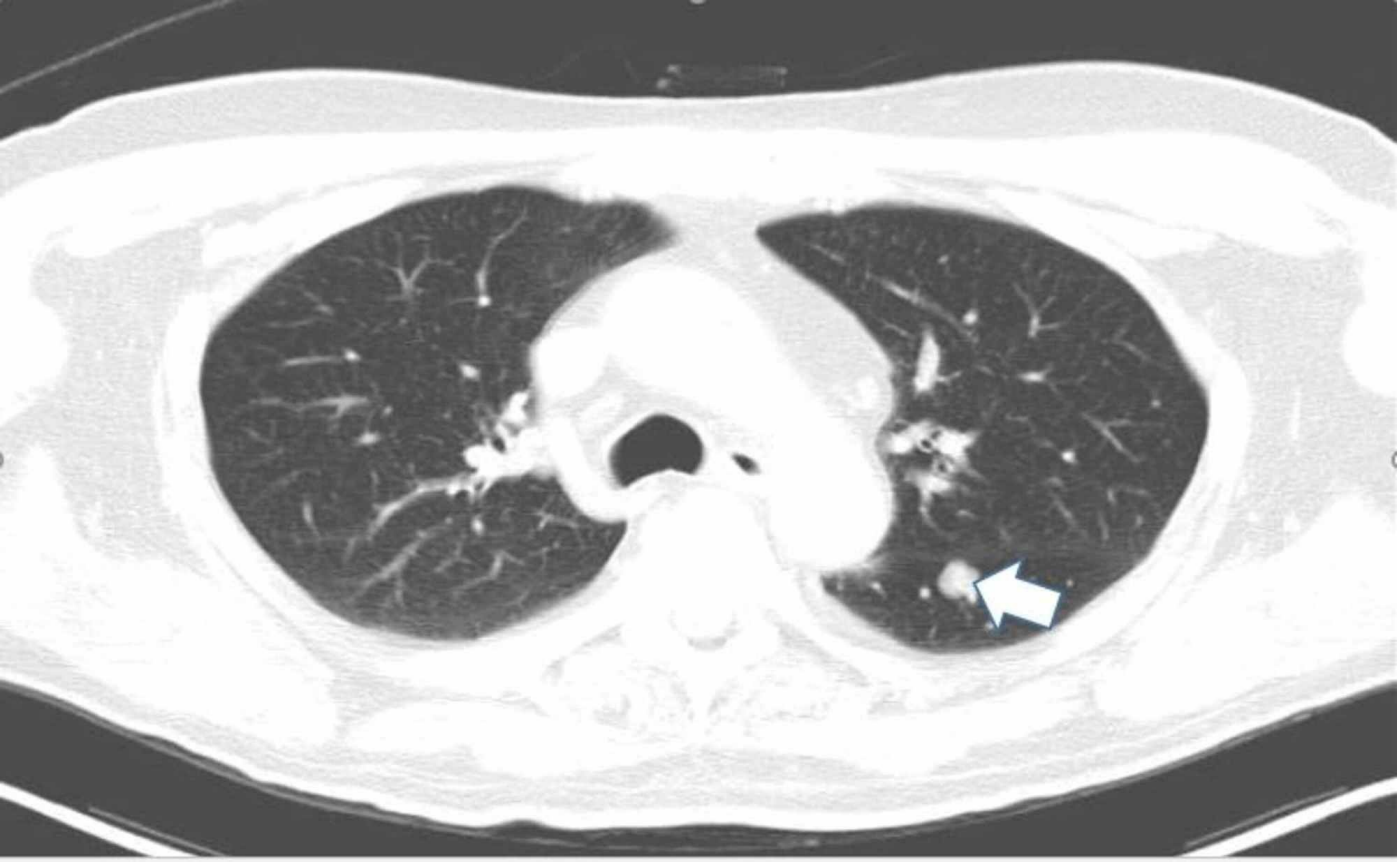
CT scan of the lung in an axial view shows a 10-mm nodule in the left lower lung (white arrow) CT: computed tomography

**Figure 2:**
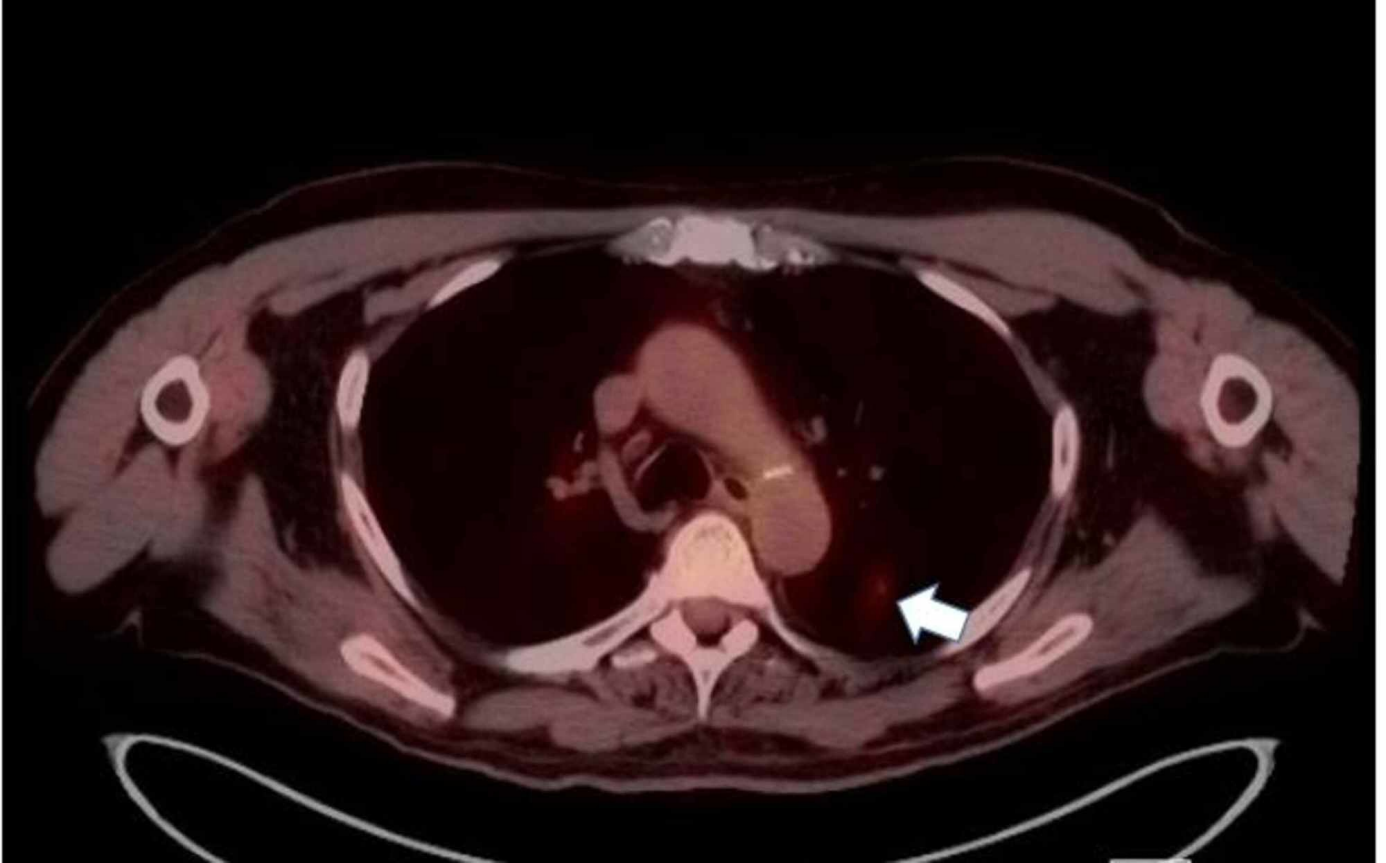
CT/PET scan of the lung in an axial view shows abnormal uptake of the 10-mm nodule in the left lower lobe of the lung (white arrow) CT: computed tomography; PET: positron emission tomography

**Figure 3:**
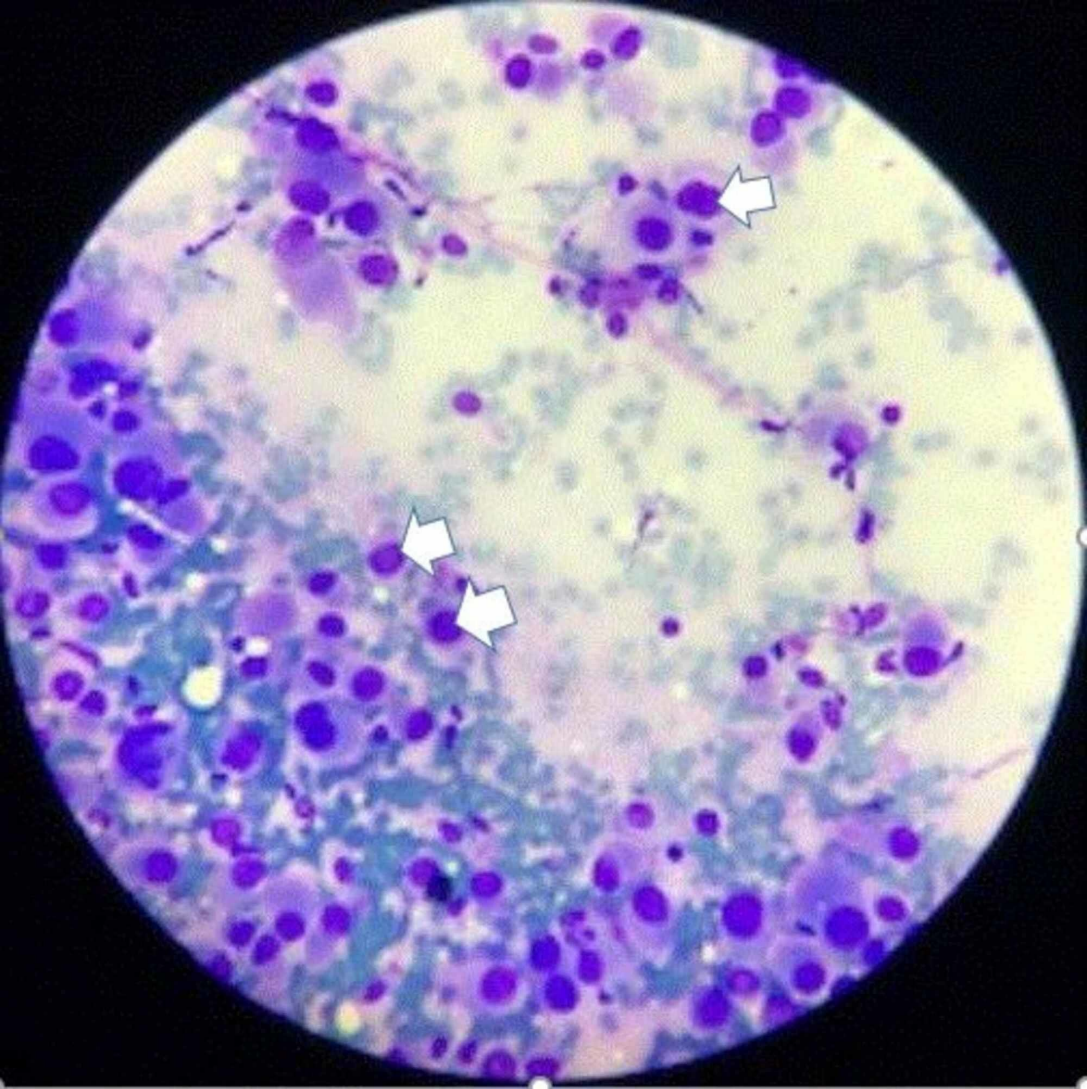
A microscopic picture shows a malignant neoplasm of plasmacytoid cells (arranged in a nested pattern with rare intranuclear inclusions as pointed by white arrows) surrounded by normal lung tissue

**Figure 4:**
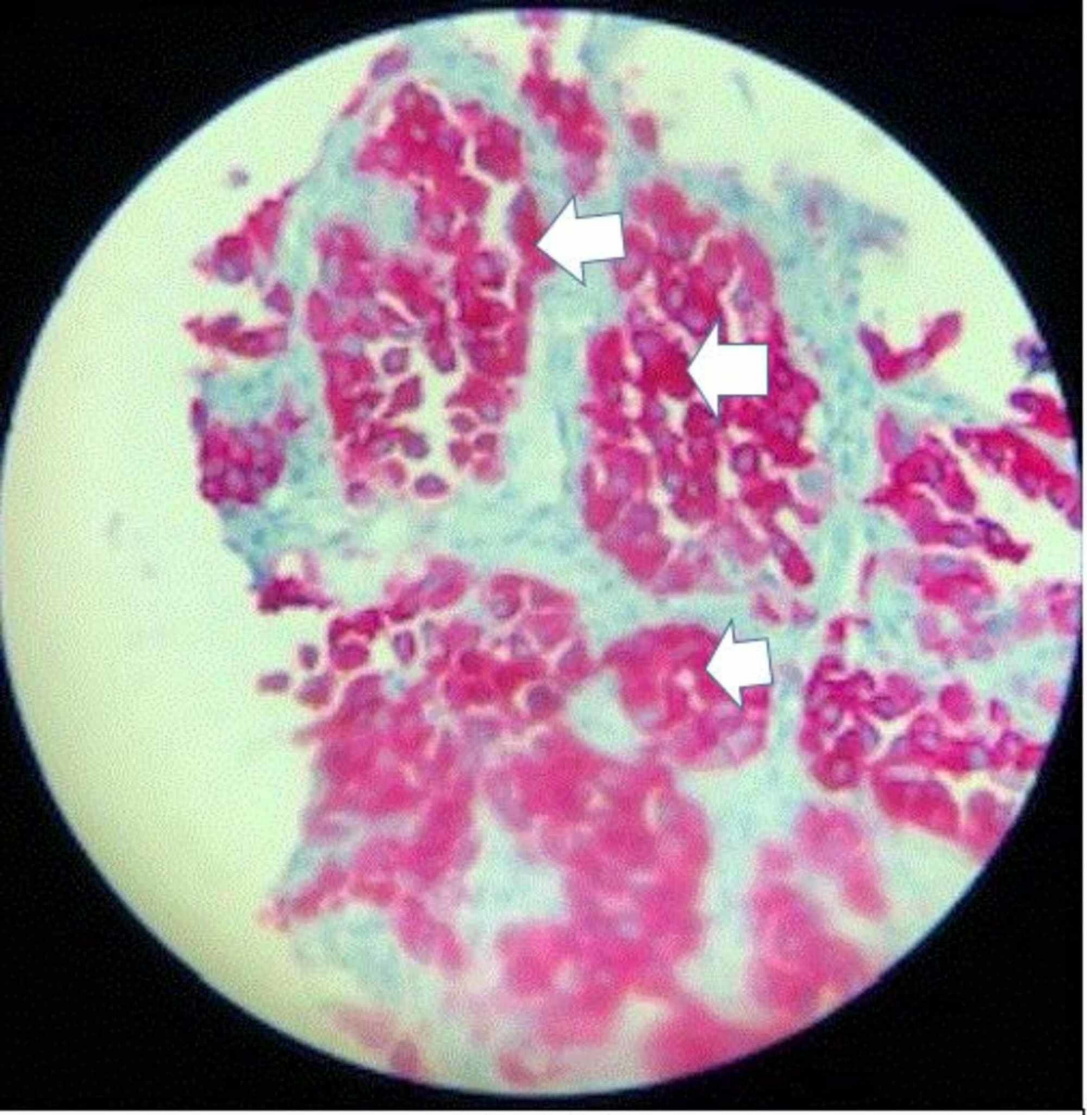
A microscopic picture shows the immunohistochemical staining MART-1, strongly immunoreactive, confirming that the nested plasmacytic neoplasm is consistent with metastatic melanoma

A 76-year-old man with a past medical history of prostate cancer, paroxysmal atrial fibrillation, and recurring pneumonia was found to have a 10-mm nodule in the left lower lung without hilar or mediastinal lymphadenopathy on CT of the chest with IV contrast (Figure 1). CT/PET scan showed abnormal uptake in the lung nodule (Figure 2) and grade II cervical lymphadenopathy. CT-guided biopsy of the left lower lung was performed. Histopathology showed a malignant neoplasm of plasmacytoid cells (Figure 3). A panel of immunohistochemical stains was performed to further evaluate the lesion. The tumor cells were strongly and diffusely immunoreactive for the melanocytic marker MART-1 (Figure 4). However, stains for AE1/AE3, CK7, TIF-1, Napsin, P63, PSA, and PASP were negative. The immunohistochemical results confirmed a diagnosis of metastatic melanoma of unknown primary origin (MUP) to the cervical lymph nodes (LNs) and lungs. The patient had just finished four cycles of ipilimumab when he presented to our clinic complaining of fatigue, generalized weakness, dizziness, nausea, abdominal pain, and a 10-pound weight loss. The patient denied any fever, chills, chest pain, shortness of breath, or diarrhea. He denied any smoking or drug-use history. Vital signs included a blood pressure of 98/60 mmHg, heart rate of 92 bpm, respiratory rate of 20 bpm, and oxygen saturation of 92% on room air. The physical exam was unremarkable except for a slightly enlarged left cervical LN, dark pigmentation of the lips and gingiva, and sinus tachycardia without abnormal heart sounds or murmurs. The complete blood count was within normal limits.

The labs before starting ipilimumab were as follows - sodium (Na): 140 mEq/L (normal range: 135-145 mEq/L), potassium: (K) 3.6 mEq/L (normal range: 3.5-5.2 mEq/L), chloride (Cl): 105 mEq/L (normal range: 96-106 mEq/L), carbon dioxide (CO2): 28 mEq/L (normal range: 23-29 mEq/L), blood urea nitrogen (BUN): 19 mg/dL (normal range: 6-20 mg/dL); creatinine (Cr): 1.3 mg/dL (normal range: 0.8-1.2 mg/dL); albumin: 3.9 g/dL, and glucose: 110 mg/dL (normal range: 64-100 mg/dL); morning cortisol: 17 μg/dL (normal range: 5-25 μg/dL), adrenocorticotropic hormone (ACTH): 14 pg/mL (normal range: <80 pg/mL), thyroid-stimulating hormone (TSH): 2.4 μU/mL (normal range: 0.4-5 μU/mL), and free thyroxin: 1.2 ng/dL (normal range: 0.8-2.8 ng/dL).

The labs after four cycles of ipilimumab were as follows - Na: 131 mEq/L, K: 4.1 mEq/L, Cl: 87 mEq/L, CO2: 27 mEq/L, BUN: 6 mg/dL, Cr: 1.1 mg/dL, albumin: 3.7 g/dL, and glucose: 89 mg/dL; morning cortisol: 5 μg/dL, ACTH: 120 pg/mL, TSH: 5 μU/mL, free thyroxin: 0.9 ng/dL, testosterone: 437 ng/dL (normal range: 270-1,070 ng/dL), follicle-stimulating hormone (FSH): 3.5 mIU/mL (normal range: 1.5-12.4 mIU/mL), luteinizing hormone (LH): 6.8 mIU/mL (normal range: 1.24-7.8 mIU/mL), prolactin: 11 ng/mL (normal range: 2-18 ng/mL). Aldosterone was undetectable, and renin was 31 ng/mL/h (normal range for normal sodium diet: 0.6-4.3 ng/mL/h). The patient's HbA1C was 5.7%, and blood culture showed no growth. Urinalysis showed no abnormality and procalcitonin was negative. After the administration of 250 μg intravenous (IV) cosyntropin, cortisol was found to be 6.8 μg/dL at 30 mins, and 10.2 μg/dL at 60 mins, confirming the diagnosis of PAI.

**Figure 5:**
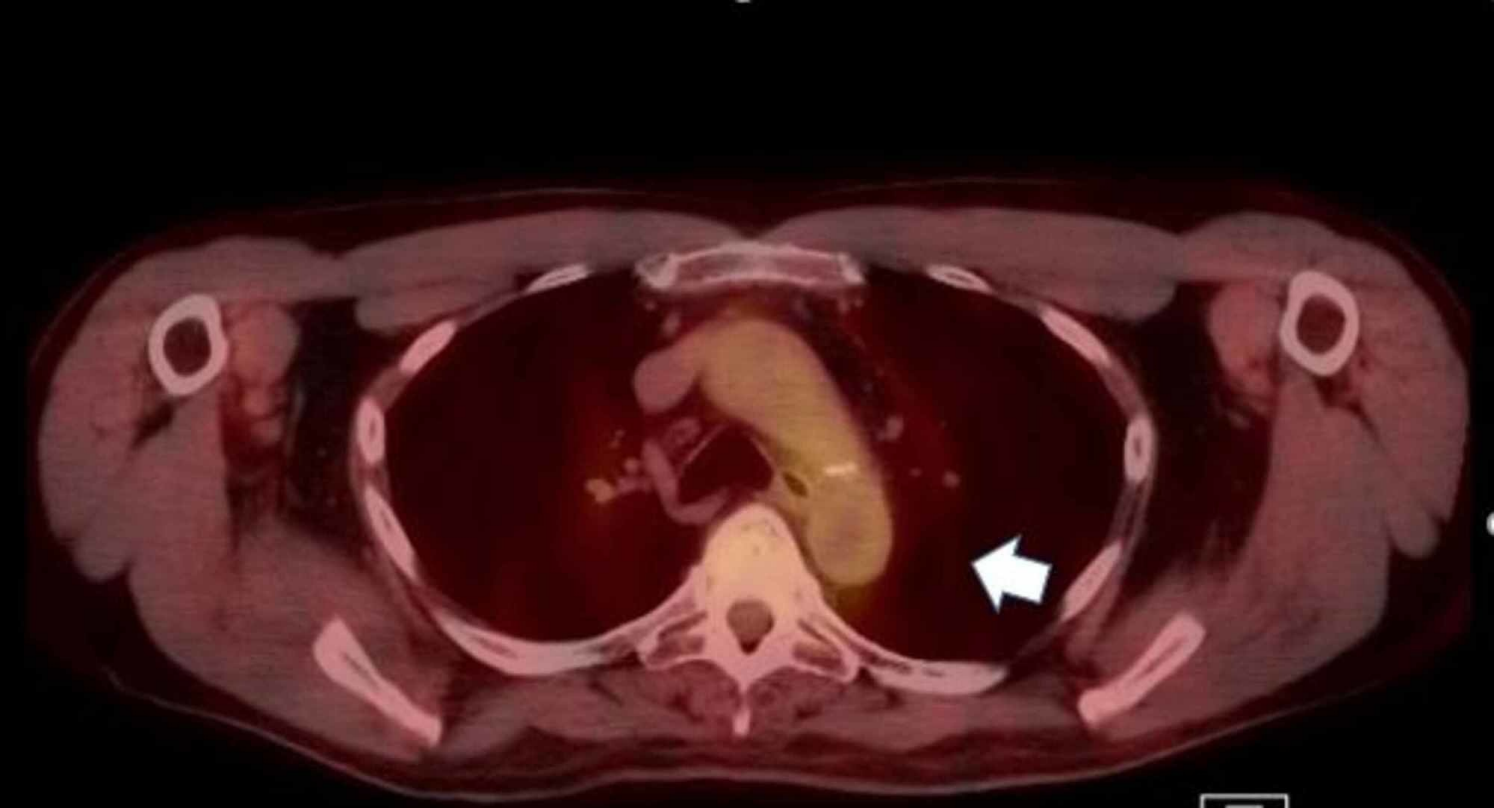
CT/PET scan of the lung in an axial view shows a complete resolution of the left lower nodule (white arrow) CT: computed tomography; PET: positron emission tomography

He was diagnosed with grade 3 toxicity of ipilimumab and, given his severe symptoms limiting his daily activities, was hospitalized. The patient was immediately started on IV fluid resuscitation and hydrocortisone 100 mg IV bolus, followed by 50 mg IV every six hours. A CT of the abdomen and pelvis with and without IV contrast revealed no evidence of intraabdominal or pelvic pathology. MRI of the brain with and without contrast revealed no evidence of hypophysitis and no evidence of intracranial metastatic disease. Symptomatic improvement was seen at 24 hours. He was discharged with a four-day taper of hydrocortisone as per the recommendations of the endocrinologist. On his follow-up at the endocrinology clinic, his symptoms continued to improve. A repeat cosyntropin stimulation test showed cortisol levels within normal limits. The patient was started on a second checkpoint inhibitor, nivolumab, for 16 cycles of treatment while being followed up by an endocrinologist to monitor any side effects. A repeat CT/PET FDG 12 months later showed complete disappearance of the left lung nodule, indicating tumor remission (Figure 5).

## Discussion

The field of cancer treatment is rapidly evolving. There have been exponential advances in the development of immunotherapy and gene therapy targeted towards specific proteins in oncologic pathways. Ipilimumab, a monoclonal antibody directed against CTLA-4, is a novel agent that increases overall survival in metastatic melanoma. It has demonstrated that targeting an immune checkpoint can improve outcomes in patients with cancer [1]. The antibodies that target PD-1 (e.g., nivolumab, pembrolizumab, atezolizumab, avelumab, and durvalumab) are active against a broad range of malignancies and are changing the treatment landscape for cancer [2]. These agents are being used as monotherapy or in combination regimens. However, because of their targets and mechanisms of action, immune checkpoint inhibitors can cause autoimmune and inflammatory effects, termed irAEs. Affected organ systems include, but are not limited to, the skin, gastrointestinal (GI) tract, the liver, and the endocrine system. The irAEs associated with immune checkpoint inhibitors are generally consistent across tumor types [3]. Toxicities are reversible in most cases by using established treatment algorithms, except for those that affect the endocrine system [3,4].

MUP is defined by the presence of melanoma in distant subcutaneous sites, LNs, or visceral organs without an obvious cutaneous, ocular, or mucosal primary site. It comprises 3.2% of all new melanoma diagnoses; it is more common in men and has a peak incidence in the fourth and fifth decades of life. LN spread occurs more often than the involvement of subcutaneous or visceral tissues, and it most commonly affects the axillary LNs. Furthermore, the involvement of a wide variety of visceral organs has been reported with MUP, and the possibility of primary non-cutaneous melanomas occurring as a result of the malignant degeneration of ectopic melanocytes present in visceral organs, including the GI tract, should be considered [5]. MUP should be classified as American Joint Committee on Cancer (AJCC) stage III disease if it is found in LNs or subcutaneous tissue at initial presentation, or as AJCC stage IV disease if it is diagnosed in visceral organs. MUP patients have better prognoses and improved overall survival (OS) rates compared to stage-matched patients of melanoma of known primary (MKP), suggesting that immunologically mediated primary site regression may be the common underlying mechanism explaining the biological phenomenon of MUP [5].

Inflammation of the pituitary, thyroid, or adrenal glands as a result of checkpoint blockade referred to as irAEs often presents with nonspecific symptoms such as nausea, headache, fatigue, and vision changes. The incidence of endocrinopathies has been difficult to define precisely, due to variable methods of assessment, diagnosis, and monitoring in different clinical trials. The most common endocrinopathies are hypothyroidism, hyperthyroidism, and hypophysitis. As per Barroso-Sousa et al.'s systematic review and meta-analysis that included 7,551 patients in 38 randomized trials, the overall incidence of clinically significant endocrinopathies is approximately 10% of patients treated with checkpoint inhibitors [6,7]. The most critical endocrinopathy is adrenal insufficiency, which can cause dehydration, hypotension, and electrolyte imbalances (hyperkalemia, hyponatremia) and constitutes an emergency. Adrenal insufficiency is rare and has been reported in only 0.7% of patients treated in randomized clinical trials, with a median time to the onset of moderate to severe immune-related endocrinopathy of 7-20 weeks [7,8].

**Figure 6:**
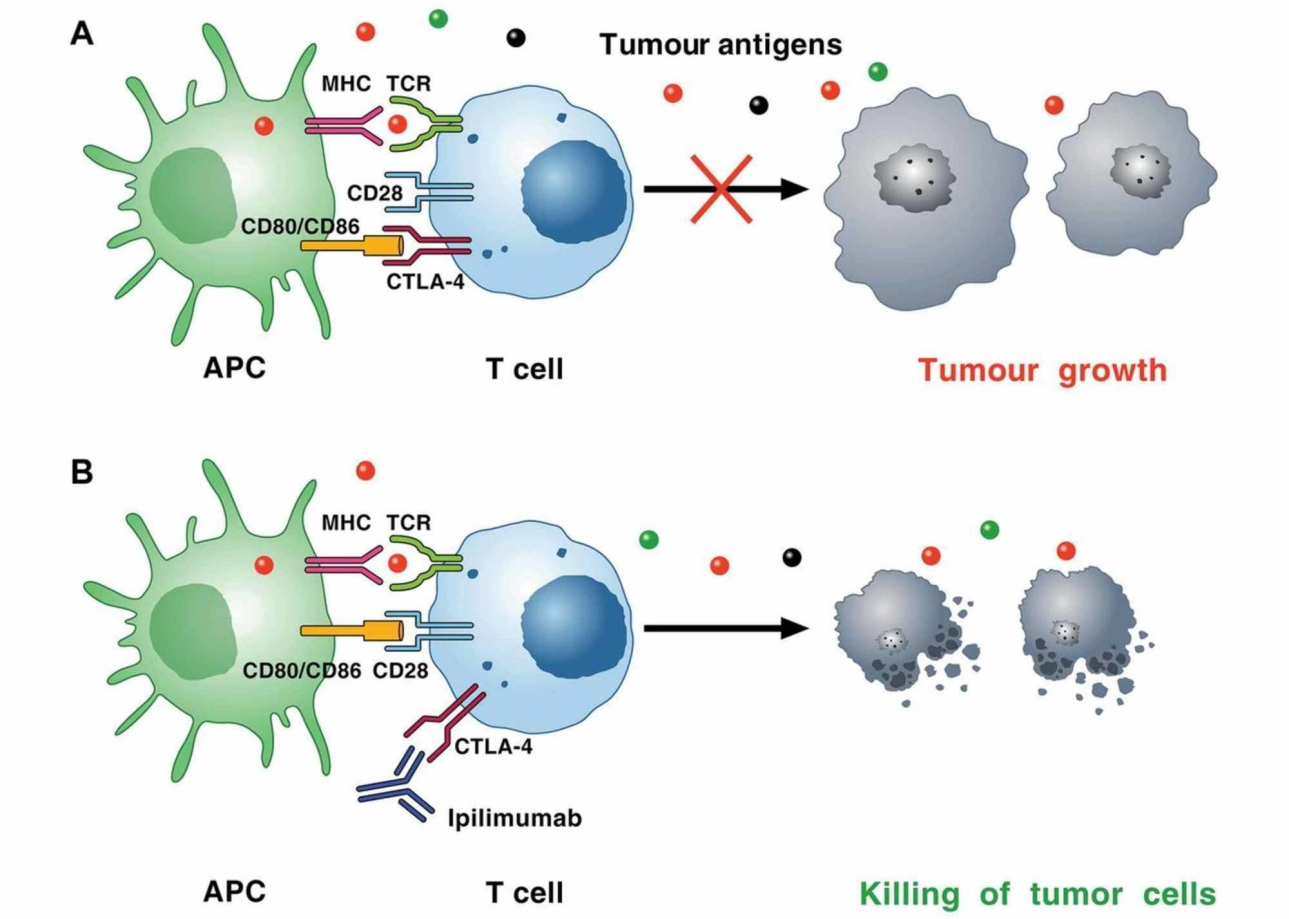
Mechanism of CTLA-4-induced immunosuppression (A): cancer cells synthesize and release neoantigens (dots of different colors) that are captured by APCs. These cells present peptides in the context of MHC I molecules/TCRs on the surface of CD8+ cytotoxic T cells within lymph nodes. APCs can also present peptides bound to MHC II molecules to CD4+ T helper cells. T cell activation on TCR signaling requires costimulatory signals transmitted via CD28, which is activated by binding to CD80, and/or CD86, on the surface of APCs. Activated T cells upregulate CTLA-4, which competes with CD28 for binding to CD80 and/or CD86. The interaction of CTLA-4 with CD80 or CD86 results in inhibitory signaling promoting tumor growth. The immunosuppressive activity of CTLA-4 is mediated by the downregulation of Th cells and the enhancement of Treg cells. (B) CTLA-4 blockade by ipilimumab results in a broad enhancement of immune responses against neoantigen expressing tumor cells, which results in the killing of tumor cells APC: antigen-presenting cell; MHC: major histocompatibility complex; CTLA-4: cytotoxic T lymphocyte-associated antigen 4; TCR: T cell receptor; Th cells: helper CD4+ T cells; Treg: regulatory T cell Courtesy of Dr. Varricchi, et al. [9]. Image reproduced with permission of BMJ Publishing Group Ltd.

Ipilimumab-associated endocrinopathies appear to be autoimmune in nature. CTLA-4 is a protein expressed on T cells and competes against CD28 to bind the co-stimulatory molecule B7 on antigen-presenting cells (APC). CTLA-4 does not produce a stimulatory signal, thereby counteracting the activating CD28/B7 and T-cell receptor/major histocompatibility complex (MHC) pathways, inhibiting T-cell function. Ipilimumab blocks CTLA-4, thereby supporting the activation and proliferation of effector T cells and reducing immunosuppressive regulatory T cells as shown in Figure 6 [9]. The blockade of CTLA-4 leads to loss of tolerance to self-antigens and causes autoimmunity. As per Caturegli P et al., CTLA-4 is present in the normal pituitary cells, and patients who are treated with ipilimumab can develop autoantibodies against CTLA-4 expressed on pituitary cells, which cause complement activation with C3d and C4d deposition and inflammatory cascade, leading to the development of hypophysitis [10].

Routine laboratory testing for TSH is recommended prior to treatment with checkpoint inhibitors. Additional testing for ACTH, cortisol, growth hormone, prolactin, LH, FSH, and testosterone is indicated for suspected immune-mediated endocrinopathies. If needed, pituitary gland imaging may be warranted with MRI of the brain [11].

Adrenal insufficiency (either primary or secondary to hypopituitarism) is characterized by hypotension, dehydration, hyponatremia, and hyperkalemia that may mimic sepsis syndrome. This case highlights the clinical barriers that occur while evaluating patients on checkpoint inhibitors. Fatigue is a very common complaint (15-28%) and is not a symptom that would frequently prompt evaluation for PAI [12]. However, in combination with weight loss, nausea, and lightheadedness, a diagnosis of PAI should be considered, particularly due to its life-threatening nature and need for urgent medical attention. Hyponatremia and hyperkalemia are supportive labs that should prompt high suspicion. If adrenal insufficiency is suspected, ACTH and cortisol levels need to be checked, and endocrinology consultation should be considered [13,14]. Adrenal insufficiency most often requires hospitalization and management with IV fluids and corticosteroids after sepsis is ruled out. Hydrocortisone should be initiated with a 100 mg IV bolus that should be given immediately, followed by 50 mg IV every six hours for the first 24 hours. After the initial 24 hours, the dose may be gradually tapered; once the patient is stable, oral maintenance dosing may be resumed. Immune checkpoint inhibition may be continued once the symptoms resolve [15].

## Conclusions

With the increasing use of checkpoint inhibitors like ipilimumab and nivolumab in cancer treatment, we expect irAEs to become an increasingly common problem encountered by clinicians. While the most common endocrinopathies reported with immune checkpoint inhibitor therapy are hypophysitis and hypothyroidism, our case involved a PAI that responded well to steroid replacement. Moreover, PAI is more likely than secondary adrenal insufficiency to be life-threatening. Given the unique nature of adverse events produced by immune checkpoint inhibitors, a multidisciplinary team approach is required to effectively manage this patient population so as to minimize the morbidity arising from agent-related toxicity.
